# Carotid plaque rather than intima-media thickness as a predictor of recurrent vascular events in patients with acute ischemic stroke

**DOI:** 10.1186/s12947-017-0110-y

**Published:** 2017-07-24

**Authors:** Hyun Ju Yoon, Kye Hun Kim, Hyukjin Park, Jae Yeong Cho, Young Joon Hong, Hyung Wook Park, Ju Han Kim, Youngkeun Ahn, Myung Ho Jeong, Jeong Gwan Cho, Jong Chun Park

**Affiliations:** 1 Department of Cardiovascular Medicine, Chonnam National University Hospital, 42 Jaebong-ro, Donggu, Gwangju, 501-757 South Korea; 2 Translational Research Center on Aging, Chonnam National University Hospital, 42 Jaebong-ro, Donggu, Gwangju, 501-757 South Korea

**Keywords:** Carotid artery, Plaque, Intima-media thickness, Stroke

## Abstract

**Background:**

To investigate the impacts of carotid plaque and intima-media thickness (IMT) on future vascular events (VEs) in the patients with acute ischemic stroke.

**Methods:**

A total of 479 consecutive Korean patients with acute ischemic stroke were divided into 2 groups according to development of VEs; VE group (65.4 ± 10.9 years) vs no VE group (62.8 ± 13.2 years). VEs were defined as the development of recurrent stroke, coronary events, peripheral arterial disease, and death. Clinical, laboratory, and imaging findings were compared between the groups.

**Results:**

During 105.5 ± 29.0 months of follow up, VEs were developed in 142 patients (29.6%). In univariate analysis, VEs were significantly associated with age, gender, diabetes, renal function, lipid levels, left ventricular function, carotid plaque or IMT. In multivariate analysis, the presence of carotid plaque, diabetes, renal function and male gender were independent predictors of future VEs in the patients with ischemic stroke, but carotid IMT was not a predictor of future VEs. Event free survival was significantly lower in patients with carotid plaque than without carotid plaque on Kaplan-Meier analysis (log rank *p* < 0.001).

**Conclusion:**

The present study demonstrated that diabetes, impaired renal function, male gender, and the presence of carotid plaque rather than IMT were independent predictors of future VEs in Korean patients with acute ischemic stroke. Active medical management and careful monitoring for the development of recurrent VEs are strongly recommended in patients with acute ischemic stroke and carotid plaque.

## Background

Atherosclerotic cardiovascular disease (CVD) is a major cause of mortality and morbidity in worldwide. Carotid atherosclerosis is not only a marker of systemic atherosclerosis but also a predictor of ischemic cerebrovascular disease [1]. Carotid ultrasound is an efficient, relatively inexpensive, highly reproducible method to evaluate atherosclerotic change of the carotid artery by measuring the presence of plaques or intima-media thickness (IMT) of the carotid artery. The increased carotid IMT and the presence of carotid plaques are well established predictors of CVD and ischemic stroke [2–5]. Because intima-media thickening or plaques of the carotid artery may reflect different biological aspects of atherosclerotic process, the significance of carotid plaque or IMT in the prediction of atherosclerotic CVD or ischemic stroke may be different. According to the results of a recent meta-analysis, the measurement of carotid plaque seems to be a superior method than carotid IMT in predicting the development of CVD [6, 7]. In contrary to carotid plaque, furthermore, the association between carotid IMT and CVD has been questioned in some studies [8, 9].

After a first attack of stroke, secondary prevention for future vascular events (VEs) including recurrent stroke is very important. Major hemispheric stroke, ischemic stroke, and atrial fibrillation have been suggested as clinical predictors of recurrent stroke after an index stroke [10]. Recent studies have also suggested that carotid IMT or plaque can be a useful imaging marker for stroke recurrence [11–14]. However, the comparison between carotid IMT and plaque on future VEs including stroke recurrence after an index stroke has been poorly studied. We hypothesized that the significance of carotid IMT and plaque on future VEs would be different after a first attack of stroke of ischemic etiology. Therefore, the aim of this study was to investigate the impacts of carotid IMT and plaque on future VEs in the patients with acute ischemic stroke.

## Methods

### Study design and population

The present study is a single center, retrospective observational study, and the study protocol was approved by the Institutional Review Board of our institution (No = 2010–05-092).

From 2007 to 2008, a total of 2607 Korean patients were diagnosed as acute stroke. After excluding 2128 patients, a total of 479 patients with ischemic stroke who had baseline echocardiography and carotid ultrasound at admission were finally enrolled and divided into 2 groups according to the development of VE: VE group (*n* = 142, 65.42 ± 10.9 years, 99 males) vs no VE group (*n* = 337, 62.77 ± 13.2 years, 166 males). The reasons of exclusion were as follows; 1) no baseline echocardiography or carotid ultrasound study (*n* = 837), 2) previous history of stroke (*n* = 361), 3) cardio-embolic stroke including atrial fibrillation, mechanical valve, or mitral stenosis (*n* = 276), 4) transient ischemic attack (*n* = 269), 5) hemorrhagic stroke (*n* = 260), 6) cryptogenic stroke with confirmed patent foramen ovale (*n* = 125). After discharge from an index stroke, the study subjects underwent clinical follow up at out-patient clinic for 105.5 ± 29.0 months, and follow up information including VEs were obtained from medical records.

### Definition of stroke and VEs

According to the updated definition of stroke of the current guideline, ischemic stroke was diagnosed by the combination of symptoms and/or signs of typical neurological dysfunction and imaging evidence of central nervous system infarction. Therefore, ischemic stroke was defined as an episode of neurological dysfunction caused by focal cerebral, spinal, or retinal infarction on imaging studies [15].

VEs were defined as the development of recurrent stroke, coronary events, peripheral arterial disease, and cardiovascular death during the study period in the present study. Recurrent stroke was defined as the development of a new focal neurologic deficit or new deterioration of a previous deficit accompanied by a de novo imaging evidence of brain infarction, but the development of transient ischemic attack or hemorrhagic transformation of the previous infarct lesion was excluded in the present study. Coronary event was defined as the development of obstructive atherosclerotic coronary artery disease (% diameter stenosis >70%) or myocardial infarction demonstrated by conventional coronary or cardiac CT angiography. Peripheral arterial disease was defined as the development of a narrowing of the arteries other than those that supply the heart or the brain with an ankle brachial index <0.9.

### Laboratory tests

Routine laboratory study was performed as soon as possible after admission, and blood samples to assess the serum lipid profile and glucose were obtained in the next morning after fasting more than 9 h. C-reactive protein (CRP) was measured by the immunoturbidimetric CRP-Latex (II) assay using an Olympus 5431 auto analyzer (Olympus America Inc., Melville, NY, USA).

### Carotid ultrasound examination

Carotid ultrasound examination was performed on both common carotid arteries (CCA) and internal carotid arteries (ICA) using a 10 MHz linear probe (Vivid 7, GE Vingmed Ultrasound, Horten, Norway) according to the current guideline [16, 17]. With the subject in the supine position and with slight hyperextension of the neck, the CCA and carotid bulb were identified.

Carotid arteries were examined bilaterally in the transversal and longitudinal planes. Following short-axis 2D image acquisition of the CCA, long-axis B-mode ultrasound images were acquired for the subsequent measurements. After placing a region of interest in the far wall of the CCA, the mean IMT was estimated in a region free of atherosclerotic plaques by using semi-automated vessel-wall detection software, AutoIMT® by GE Healthcare [18]. Averaged IMT values of the left and right CCAs were subsequently used in all analyses. Carotid IMT is defined as a double-line pattern visualized by echo 2D on both walls of the CCA in a longitudinal view. Two parallel lines (leading edges of two anatomical boundaries) form it. Mean IMT was computed from 80 to 120 measurements over a 10 mm span ending 5 mm proximal to the transition between the CCA and bulb regions. Intra- and inter-operator coefficients of variation were 2.8% and 3.0%, respectively, and intra- and inter-operator intra-class correlations were both 0.97. Plaque was defined as a protrusion of the vessel wall into the arterial lumen of at least 0.5 mm, with an IMT 50% that of the surrounding sites or an IMT > 1.5 mm as measured from the media-adventitia interface to the intima lumen interface. Regardless of locations, the presence of any plaque in CCA or bulb or ICA was considered as the presence of carotid plaque in the present study. First of all, the presence or absence of carotid plaques were evaluated and then texture were classified into soft, mixed, and calcified based on echogenicity. In the case of multiple plaques, we measured maximal protruding diameter from carotid wall. On a longitudinal two-dimensional ultrasound image of the carotid artery, the anterior (near) and posterior (far) walls of the carotid artery appear as two bright white lines separated by a hypoechogenic space. End-diastolic images were frozen, and the far wall IMT was identified as the region between the lumen-intima interface and the media-adventitia interface using semiautomatic method. It contained CCA, bulb and proximal ICA plaques. Peak systolic and end diastolic carotid flow velocity was measured by pulse wave Doppler on the CCA and the ICA [19].

### Echocardiographic examination

Echocardiographic images from various echocardiographic windows were obtained by using a digital ultrasonographic equipment system (Vivid 7, GE Vingmed Ultrasound, Horten, Norway). Digital cine loops were obtained for subsequent offline analysis. All of the data were analyzed by using the computerized offline software package (EchoPAC PC 6.0.0, GE Vingmed Ultrasound, Horten, Norway). Routine echocardiographic examinations were performed in accordance with the recommendation of the current guideline [20]. Ejection fraction were measured by using Simpson’s biplane method. The intra-observer and inter-observer variabilities of Simpson’s method were 4% ± 5% and 5% ± 4% (absolute difference divided by the mean measurement value). The early (E) and late diastolic velocities (A) of the mitral inflow were measured by using pulsed wave Doppler from the apical 4-chamber view, with the sample volume positioned at the tip of the mitral leaflets. The early (*e*′), late diastolic (*a*′), and systolic velocities of the mitral septal annulus were measured by using tissue Doppler imaging in the apical four chamber view.

### Statistical analysis

The statistical Package for Social Sciences (SPSS) for Windows, version 18.0 (Chicago, Illinois, USA) was used for statistical analysis. Data are presented as percents or mean ± standard deviation. The differences of the categorical variables were evaluated by Chi-square test, and the continuous variables were compared using independent *t* test. The event-free survival rate was evaluated by using the Kaplan-Meier analysis, and the event rates were compared using the log-rank test. To identify the independent predictor of long term vascular events, multivariate logistic regression analysis was applied to the significant variables in univariate analysis. A *p*-value less than 0.05 was considered as statistically significant.

## Results

### Baseline characteristics

Baseline characteristics are summarized in the Table 1. Age was older and male gender was more prevalent in VE group than in no VE group. Diabetes mellitus and prior history of cerebrovascular accident were more frequent in VE group than in no VE group. Other baseline characteristics were not different between the groups.

**Table 1:** Baseline clinical characteristics of the patients

	VE (*n* = 142)	No VE (*n* = 337)	*P* value
Age (years)	65.4 ± 10.9	62.7 ± 13.4	0.035
Male (%)	99 (70.2%)	166 (50%)	<0.001
Height (cm)	162.2 ± 9.1	161.5 ± 839	0.525
Weight (kg)	63.7 ± 9.6	63.4 ± 11.3	0.825
BMI (kg/m^2^)	25.5 ± 14	24.9 ± 9.1	0.655
Hypertension (%)	86 (61.8%)	159 (54.1%)	0.077
Diabetes (%)	53 (38.1%)	62 (21.0%)	<0.001
Dyslipidemia (%)	92 (67.1%)	202 (68.7%)	0.414
Smoking (%)	31 (22.3%)	59 (20.1%)	0.664
Territory infarction (%)	54 (38.8%)	127 (41.7%)	0.317
SBP (mmHg)	135.4 ± 22.7	133.1 ± 19.5	0.277
DBP (mmHg)	82.3 ± 14.6	81.9 ± 13.3	0.845
Pulse pressure (mmHg)	53.9 ± 16.7	51.5 ± 13.6	0.106

*BMI* body mass index, *CVA* cerebrovascular accident, *SBP* systolic blood pressure, *DBP* diastolic blood pressure

### Laboratory findings and discharge medication

Laboratory findings are summarized in the Table 2. The levels of serum glucose, hemoglobin A1c, and creatinine were significantly higher, whereas the levels of total and low density lipoprotein cholesterol were significantly lower in VE group than in no VE group. There was no significant difference in discharge medication between groups (Table 3).

**Table 2:** Laboratory findings of the patients

	VE (*n* = 142)	No VE (*n* = 337)	*P* value
WBC (/mm^3^)	7498 ± 4545	7383 ± 4746	0.814
Hb (g/dL)	13.0 ± 1.9	13.5 ± 2.7	0.095
TC (mg/dL)	179.1 ± 39.0	187.2 ± 37.1	0.044
TG (mg/dL)	129.2 ± 63.0	127.5 ± 70.9	0.819
LDL-C (mg/dL)	110.4 ± 35.1	124.2 ± 42.3	0.002
HDL-C (mg/dL)	45.7 ± 14.8	63.2 ± 26.3	0.456
LP(a) (mg/dL)	32.2 ± 30.4	38.2 ± 12.1	0.601
Glucose (g/dL)	146.1 ± 64.4	129.8 ± 55.8	0.011
HbA1c (%)	6.45 ± 1.1	6.27 ± 1.3	0.017
Creatinine (mg/dL)	1.10 ± 0.98	0.83 ± 0.363	0.001
CRP (mg/dL)	1.68 ± 3.5	1.04 ± 2.1	0.057
Homocystein (umol/L)	12.0 ± 5.2	12.0 ± 10.3	0.993

*WBC* white blood cell, *Hb* hemoglobin, *TC* total cholesterol, *TG* triglyceride, *LDL-C* low density lipoprotein-cholesterol, *HDL-C* high density lipoprotein-cholesterol, *LP* lipoprotein, *HbA1c* glycosylated hemoglobin, *CRP* C reactive protein

**Table 3:** Prescribed medications of the patients

	VE (*n* = 142)	No VE (*n* = 337)	*P* value
Aspirin (%)	50 (35.2)	107 (31.7)	0.416
Plavix (%)	49 (34.5)	106 (31.4)	0.161
Warfarin (%)	28 (19.7)	54 (16.0)	0.321
ACEI or ARB (%)	56 (39.4)	128 (37.9)	0.307
Beta blocker (%)	30 (21.1)	69 (20.4)	0.238
CCB (%)	53 (37.3)	102 (30.2)	0.064
Statin (%)	42 (29.5)	81 (24.0)	0.308

*ACEI* angiotensin converting enzyme, *ARB* aldosterone receptor blocker, *CCB* calcium channel blocker

### Echocardiographic findings

Echocardiographic findings are summarized in the Table 4. Left ventricular end-systolic dimension was larger, whereas ejection fraction was decreased in VE group than in no VE group. Other echocardiographic findings were not different between the groups.

**Table 4:** Baseline echocardiographic findings of the patients

	VE (*n* = 142)	No VE (*n* = 337)	*P* value
LVEDD (mm^2^)	48.7 ± 5.3	48.1 ± 4.5	0.174
LVESD (mm^2^)	31.8 ± 4.9	30.8 ± 4.2	0.046
IVS thickness (mm)	9.7 ± 1.7	9.7 ± 1.6	0.913
PW thickness (mm)	9.4 ± 1.3	9.4 ± 1.2	0.737
LA dimension (mm)	39.5 ± 7.6	38.4 ± 7.7	0.150
Aorta (mm)	32.5 ± 3.8	32.6 ± 7.5	0.917
EF (%)	63.4 ± 6.5	64.8 ± 6.	0.043
E (m/s)	0.62 ± 0.30	0.62 ± 0.63	0.974
A (m/s)	0.71 ± 0.16	0.70 ± 0.21	0.458
DT (msec)	213.5 ± 117	219.3 ± 88	0.607
E’ (m/s)	0.06 ± 0.06	0.07 ± 0.06	0.572
S′ (m/s)	0.09 ± 0.13	0.10 ± 0.52	0.814
E/E’	11.2 ± 7.8	10.4 ± 6.9	0.290

*LVEDD* left ventricle end diastolic dimension, *LVESD* left ventricle end systolic dimension, *LA* left atrium, *EF* ejection fraction, *E* early diastolic mitral inflow velocity, *A* late diastolic mitral inflow velocity, *DT* deceleration time, *E*’: early diastolic velocity of mitral septal annulus, *S*′: systolic velocity of mitral septal annulus

### Carotid ultrasound findings

Carotid ultrasound findings are summarized in the Table 5. Carotid plaques were found in 181 cases (37.8%) out of 479 patients with stroke. Regardless of location, the presence of CCA, bulb, or ICA plaque was significantly frequent in VE group than in no VE group. Significant ICA stenosis (>50%) was not different between the groups. Carotid revascularization was performed only in 2 patients of VE group and 2 patients of no VE group. IMT of the left CCA and mean IMT of the both CCA were thicker in VE group than in no VE group, but IMT of the right CCA was not different between the groups. The size and types of carotid plaque and blood flow variables of the CCA were not different between the groups.

**Table 5:** Carotid ultrasound findings of the patients

	VE (*n* = 142)	No VE (*n* = 337)	*P* value
Plaque, CCA (%)	17 (11.9)	22 (6.5)	0.038
Plaque, bulb (%)	50 (35.2)	91 (27.0)	0.046
Plaque, ICA (%)	22 (15.4)	32 (9.5)	0.044
Plaque, any site (%)	66 (46.5)	114 (33.8)	0.006
Maximal plaque size (mm)	2.4 ± 0.6	2.5 ± 0.9	0.729
Types of plaque			
Soft (%)	31 (21.8)	57 (16.9)	0.121
Calcified (%)	10 (7.0)	13 (3.9)	0.098
Mixed (%)	25 (17.6)	44 (13.1)	0.454
RCCA IMT(mm)	0.82 ± 0.23	0.78 ± 0.22	0.061
LCCA IMT (mm)	0.86 ± 0.23	0.81 ± 0.22	0.043
Mean CCA IMT (mm)	0.84 ± 0.21	0.79 ± 0.20	0.022
RCCA PSV(cm/s)	47.9 ± 22.3	49.9 ± 22.3	0.373
LCCA PSV (cm/s)	51.4 ± 25.7	52.8 ± 25.3	0.609
RCCA EDV (cm/s)	13.4 ± 6.7	14.5 ± 7.5	0.958
LCCA EDV (cm/s)	13.6 ± 7.7	15.6 ± 8.1	0.124
ICA stenosis >50% (%)	9 (6.3)	11 (3.2)	0.102
Carotid revascularization (%)	2 (1.4)	2 (0.6)	0.343

*RCCA* right common carotid artery, *LCCA* left common carotid artery, *ICA* internal carotid artery, *Rt* right, *Lt* left, *CA* carotid artery, *IMT* intima-media thickness, *PSV* peak systolic velocity, *EDV* end diastolic velocity

### VEs and predictors of VEs during clinical follow up

During 105.5 ± 29 months of clinical follow up, VEs were developed in 142 patients (29.6%); recurrent stroke in 73 patients (15.2%), coronary events in 57 patients (11.9%), peripheral arterial disease in 16 patients (3.3%), and death in 9 patients (1.9%).

Multivariate analyses to identify independent predictors of VEs were performed, and the results are summarized in the Table 6. Diabetes, renal function, male gender, and the presence of carotid plaque in any site were independent predictors of future VEs in patients with acute ischemic stroke on multivariate analysis. However, carotid IMT was not an independent predictor of future VEs. The risk of future VEs was greatest in acute ischemic stroke patients with both carotid plaque and diabetes. In subgroup analysis, carotid IMT was not an independent predictor of future VEs in stroke patients without carotid plaque.

**Table 6:** Predictors of vascular events by multivariate analysis

	RR	CI	*P* value
Age	1.018	1.000–1.036	0.051
Male	2.255	1.474–3.449	<0.001
DM	2.061	1.1311–3.239	0.002
Creatinine	1.969	1.205–3.217	0.007
LVESD	1.030	0.982–1.081	0.228
EF	0.983	0.952–1.015	0.290
Plaque, CCA	1.947	1.000–3.790	0.050
Plaque, bulb	1.469	0.965–2.236	0.073
Plaque, ICA	1.749	0.946–3.015	0.060
Plaque, any site	1.699	1.139–2.533	0.027
LCCA IMT	1.696	0.692–4.158	0.248
RCCA IMT	1.766	0.719–4.339	0.215
Mean CCA IMT	2.207	0.800–6.091	0.126
DM + plaque	3.779	2.101–6.796	<0.001

*RR* relation risk, *CI* confidence interval, *DM* diabetes mellitus, *LVESD* left ventricle end systolic dimension, *EF* ejection fraction, *CCA* right common carotid artery, *ICA* internal carotid artery, *Rt* right, *Lt* left, *CA* carotid artery, *IMT* intima-media thickness

### Carotid plaque and VEs

**Fig. 1:**
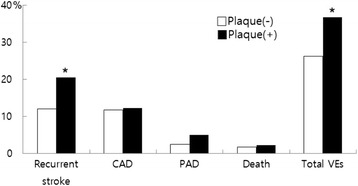
Future vascular event according to the presence of carotid plaque (* means *p* < 0.05). CAD: coronary artery disease, PAD: peripheral vascular disease, VEs: vascular events

**Fig. 2:**
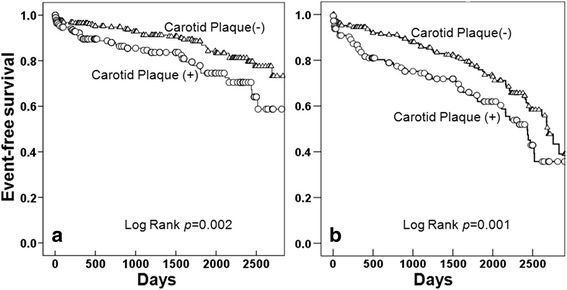
Recurrent stroke free survival (**a**) and total vascular event free survival (**b**) according to the presence of carotid plaque in patient with ischemic stroke

The presence of carotid plaque was significantly associated with recurrent stroke and total VEs, but it was not a predictor of coronary events, peripheral arterial disease and deaths (Fig. 1). On Kaplan-Meier analysis, event free survival for recurrent stroke and total VEs was significantly lower in acute ischemic stroke patients with carotid plaque than in without carotid plaque (log rank *p* < 0.001) (Fig. 2).

## Discussion

In the present study, the authors want to compare the significance between carotid IMT and plaque on future VEs including stroke recurrence after an index stroke, and the results of the present study demonstrated several clinically important findings. First, recurrent VEs are not infrequent in Korean patients with ischemic stroke after an index event, and non-cerebral VEs including coronary and peripheral artery disease comprise about a half of VEs. Second, carotid plaque rather than carotid IMT is a useful prognostic marker for the development of future VEs in Korean patients with ischemic stroke. Therefore, careful search for non-cerebral vascular diseases and active medical management for preventing recurrent stroke are strongly recommended in acute ischemic stroke patients with carotid plaque. Thirdly, the presence of carotid plaque at any sites, as a whole, was an independent predictor of future VEs, whereas the presence of plaque at CCA or bulb or ICA showed marginal significance for predicting VEs. Therefore, the present study suggested that a thorough evaluation for the presence of plaques in whole carotid arterial trees including CCA, bulb, and ICA should be performed for risk stratification in patients with acute ischemic stroke.

The risk for the development of future stroke is significantly higher in survivors of first-ever stroke than in general population [21], the risk of stroke recurrence is known to be greatest during the first week after index stroke [22]. According to a current meta-analysis, the cumulative risk of stroke recurrence is gradually increased as time goes by and is 3.1% at 30 days, 11.1% at 1 year, 26.4% at 5 years, and 39.2% at 10 years after initial stroke [23]. In the present study, recurrent stroke was developed in 15.2% during 8.7 years of clinical follow up, and the rate of stroke recurrence seems to be lower than that of a current meta-analysis [23]. The rate of stroke recurrence was 18.4% at 5 years in the retrospective cohort study of Sun et al. [24] and 19.8% at years in the study of Lee et al. [25], and the recurrence rate of these studies were similar to that of our study. The differences of stroke recurrence might be explained by the differences in study population among the studies. In contrary to a current meta-analysis [23], the present study cannot reflect true incidence of stroke recurrence, because the present study only included stroke patients of ischemic etiology with carotid ultrasound and echocardiography studies.

Stroke recurrence is known to be associated with poor long-term clinical outcomes and quality of life [22, 26]. After a first attack of stroke, therefore, early identification of high risk group and secondary prevention for future vascular events (VEs) including recurrent stroke would be very important. Despite of high prevalence of classic risk factors such as hypertension and dyslipidemia in patients with recurrent stroke as in the study of Leoo et al., hypertension and dyslipidemia were not predictors of stroke recurrence in the present study [27]. Rather, among classic risk factors for CVD, diabetes was the only significant predictor of stroke recurrence in the present study. In addition to classic risk factors, therefore, it is suggested that other risk factors or predictors for stroke recurrence should be identified to improve clinical outcomes. The previous studies have suggested several clinical predictors for stroke recurrence, and these include major hemispheric stroke, ischemic stroke, male gender, diabetes, advanced age, and atrial fibrillation [10, 23–25]. In the present study, diabetes, renal function, and male gender were significant clinical predictors of future VEs. History of coronary artery disease, severe stenosis or occlusion of large cerebral artery, and multiple acute cerebral infarcts suggested as independent predictors of recurrent ischemic stroke or TIA within one year [28]. Several studies have suggested that carotid plaque or IMT can be used as imaging marker not only in the prediction of development of first-ever stroke [2–5] but also in the prediction of stroke recurrence [11–14], even though the significance of carotid IMT or plaque may differ [6–9]. Our previous study also demonstrated that the significance of carotid IMT is different from carotid plaque on ischemic stroke [29]. Despite of these differences, the comparison between carotid IMT and plaque on future VEs after an index stroke has been poorly studied previously. And the results of the present study demonstrated that carotid plaque than carotid IMT is a significant imaging marker for stroke recurrence. The present study also suggested that a thorough evaluation for the presence of plaques in whole carotid arterial trees including CCA, bulb, and ICA should be performed for risk stratification in patients with acute ischemic stroke. In the present study, the presence of both carotid plaque and diabetes was more strongly associated with stroke recurrence, and thus this subgroup of ischemic stroke patients should be more carefully monitored and actively managed for preventing VEs.

As a whole, the prevalence of carotid plaque was 37.8% in the present study population with ischemic stroke (8.1% in CCA, 29.4% in carotid bulb, 11.3% in ICA, respectively), and carotid bulb was also the most common site for plaque formation in this study as in previous studies [16]. In the previous studies involving Korean population, the prevalence of carotid plaque was 5.7% in general population [30] and 24.6 ~ 42.1% in patients with atherosclerotic CVD such as coronary artery disease [31], and the result of the present study also showed quite similar to those of the previous studies.

In addition to the presence of carotid plaque, the characteristics of plaques are also important for predicting stroke recurrence. The previous studies have shown that echolucent or large or mobile plaques are associated with stroke recurrence [13, 32, 33], but the echogenicity, size or mobility of carotid plaque was not associated with stroke recurrence in the present study. The reason why the characteristics of plaque were not associated with VEs in the present study is unclear, and selection bias from the retrospective nature of this study or ethnic differences might be possible explanations.

In population with ischemic stroke, future coronary or peripheral artery disease events and need for revascularization are not infrequent and the result of the present study was also demonstrated the association between stroke and systemic atherosclerotic vascular events [34, 35]. The development or identification of coronary or peripheral artery disease was also not infrequent in the present study and developed in 57 patients (11.9%) and in 16 patients (3.3%), respectively. Therefore, careful evaluation for coronary or peripheral artery disease should be performed by non-invasive coronary imaging or ankle-brachial index in patients with ischemic stroke. Although the presence of carotid plaque was a significant predictor of recurrent stroke, it was not an independent predictor of future coronary or peripheral vascular events in the present study. No association between carotid plaque and systemic vascular events might be explained by multi-factorial pathogenic mechanism of coronary or peripheral artery disease or small number of study population of the present study. Increased value of CCA IMT is known to be associated with a higher long-term risk of extracranial vascular events such as coronary or peripheral artery disease in a cohort study of Purroy et al. [36], it was not s predictor of future VEs in the present study.

### Study limitations

The present study has several potential limitations. First, the present study has all limitations of a retrospective analysis including selection bias. As discussed already, the present study only included stroke patients of ischemic etiology with carotid ultrasound and echocardiography studies. Many of the patients with ischemic stroke were excluded because of the absence of ultrasound studies, and thus the result of the present study cannot be generalized. Second, the present study did not consider the impacts of interventional therapy or medications, and these also might influence on future VEs. Third, the incidence of coronary or peripheral vascular events might be underestimated because non-cerebral systemic vascular events were not evaluated routinely and usually evaluated in symptomatic patients.

## Conclusion

In conclusion, despite these potential limitations, the results of the present study demonstrated that diabetes, impaired renal function, male gender, and the presence of carotid plaque rather than IMT were independent predictors of future VEs in Korean patients with acute ischemic stroke. Active medical management and careful monitoring for the development of recurrent VEs are strongly recommended in patients with acute ischemic stroke and carotid plaque.
